# Additional Value of PET/CT-Based Radiomics to Metabolic Parameters in Diagnosing Lynch Syndrome and Predicting PD1 Expression in Endometrial Carcinoma

**DOI:** 10.3389/fonc.2021.595430

**Published:** 2021-05-12

**Authors:** Xinghao Wang, Ke Wu, Xiaoran Li, Junjie Jin, Yang Yu, Hongzan Sun

**Affiliations:** ^1^ Department of Radiology, Shengjing Hospital of China Medical University, Shenyang, China; ^2^ Liaoning Provincial Key Laboratory of Medical Imaging Department of Radiology, Shenyang, China

**Keywords:** PD1 expression, radiomics, Lynch syndrome, endometrial cancer, 18F-FDG PET/CT

## Abstract

**Purpose:**

We aim to compare the radiomic features and parameters on 2-deoxy-2-[fluorine-*18*] fluoro-D-glucose (18F-FDG) positron emission tomography/computed tomography (PET/CT) between patients with endometrial cancer with Lynch syndrome and those with endometrial cancer without Lynch syndrome. We also hope to explore the biologic significance of selected radiomic features.

**Materials and Methods:**

We conducted a retrospective cohort study, first using the 18F-FDG PET/CT images and clinical data from 100 patients with endometrial cancer to construct a training group (70 patients) and a test group (30 patients). The metabolic parameters and radiomic features of each tumor were compared between patients with and without Lynch syndrome. An independent cohort of 23 patients with solid tumors was used to evaluate the value of selected radiomic features in predicting the expression of the programmed cell death 1 (PD1), using 18F-FDG PET/CT images and RNA-seq genomic data.

**Results:**

There was no statistically significant difference in the standardized uptake values on PET between patients with endometrial cancer with Lynch syndrome and those with endometrial cancer without Lynch syndrome. However, there were significant differences between the 2 groups in metabolic tumor volume and total lesion glycolysis (*p* < 0.005). There was a difference in the radiomic feature of gray level co-occurrence matrix entropy (GLCMEntropy; *p* < 0.001) between the groups: the area under the curve was 0.94 in the training group (sensitivity, 82.86%; specificity, 97.14%) and 0.893 in the test group (sensitivity, 80%; specificity, 93.33%). In the independent cohort of 23 patients, differences in GLCMEntropy were related to the expression of PD1 (r_s_ =0.577; *p* < 0.001).

**Conclusions:**

In patients with endometrial cancer, higher metabolic tumor volumes, total lesion glycolysis values, and GLCMEntropy values on 18F-FDG PET/CT could suggest a higher risk for Lynch syndrome. The radiomic feature of GLCMEntropy for tumors is a potential predictor of PD1 expression.

## Introduction

Endometrial cancer ranks sixth in global incidence for malignant tumors, with nearly 400 000 new cases diagnosed each year (1). Treatment with hysterectomy and bilateral salpingo-oophorectomy is the standard of care. Hormone therapy, radiotherapy, and immunotherapy may be used, depending on the individual patient’s wish to preserve her uterus or her potential for fertility (2, 3). First discovered in 1895, Lynch syndrome is known to be closely related to colorectal cancer and endometrial cancer, and accounts for 2% to 6% of the latter (4, 5). Lynch syndrome creates pathology through a mutation in the mismatch repair gene (MMR) (6), and women with Lynch syndrome have a 25% to 60% likelihood of developing endometrial cancer in their lifetime (7). In patients with Lynch’s syndrome, there are differences in treatment methods, immune infiltration and PD1 expression (8–10), survival rate (11, 12) and risk in other cancers, especially colon cancer (13).

The diagnosis of Lynch syndrome is not as easy to make as that of endometrial cancer. The Amsterdam II criteria are relatively strict, and molecular diagnosis is not something that every patient with endometrial cancer can afford. Therefore, many patients with endometrial cancer and Lynch syndrome may not be detected because of a lack of family history or molecular diagnostic results. This has a negative effect on treatment, prevention of other Lynch-associated tumors, and genetic counseling.

Radiomics, also known as computational medical imaging, involves sketching, segmentation, extraction, and quantification of medical images into quantitative data points. The basic assumption is that high-dimensional imaging data not only reflects the macrocharacteristics of the tissue, but also its cellular and molecular characteristics. The objective is to produce image-driven biomarkers as a tool for a deeper understanding of cancer biology that will better assist clinical decision making. Radiomics can be used as a supplement to biopsy for noninvasive evaluation of spatial heterogeneity and the microenvironment of tumors. If endometrial cancer is identified by its metabolic parameters or radiomic features on positron emission tomography (PET), this can serve as a rough screening tool or as a predictor for directed molecular diagnostic testing. Even if molecular testing is not available, it is of great significance for patients with endometrial cancer who have the PET parameters or radiomic features of Lynch syndrome to be able to monitor their gastrointestinal health and participate in active tumor screening. Researchers have recently found that there are differences in immune cell infiltration and PD1 receptor expression between patients with Lynch syndrome-associated endometrial cancer and those with nonsyndromic endometrial cancer (8–10). To further explore the significance of selected radiomic features, we designed a cohort study to assess the relation between these features and PD1 expression.

## Materials and Methods

### Patients

This retrospective study was approved by the review committee of our institution and was adherent to the principles and requirements of the Declaration of Helsinki. We defined Cohort 1 as 100 patients with endometrial cancer, confirmed by pathology, who underwent surgery at our hospital between January 2008 and October 2019. In cohort 1, there were no significant differences in age, pathological type, stage and other conditions between the patients with Lynch syndrome and those without Lynch syndrome, so as to ensure that no other factors could affect the experimental results. The presence or absence of Lynch syndrome was confirmed by the results of pathology and genetic testing. The criteria for study inclusion were: endometrial cancer confirmed by postoperative pathology after hysterectomy, without neoadjuvant chemotherapy or radiotherapy and without other tumors or gynecologic disease; 2-deoxy-2-[fluorine-18] fluoro-D-glucose (18F-FDG) PET/computed tomography (CT) performed within 3 weeks before surgery with negative 18F-FDG uptake; access to the complete medical record with genetic testing (including Lynch syndrome) and pathologic reports. We also established Cohort 2, comprising 23 patients with solid tumors (14). The clinical and transcriptional data for Cohort 2 were obtained from the Cancer Genome Access Program (https://portal.gdc.cancer.gov/) (15), and their 18F-FDG PET/CT images were obtained from the Cancer Imaging Archive (http://www.cancerimagingarchive.net/) (16). The transcriptome analysis data included gene-level transcriptional estimates reported as log 2(x + 1) transformed RESM standardized counts. We used the PDCD1 mRNA expression level as a proxy for PD1 receptor expression (17).

### 18F-FDG PET/CT Acquisition and Features Extraction

Patients were fasting from food and water for more than 6 hours, and their blood sugar level was controlled below 7 mmol/L. One hour after intravenous injection of 18F-FDG (GE MINItrace II; GE Healthcare, Milwaukee, WI) at 0.08 to 0.16 mci/kg, PET/CT was performed from the head to the middle of the femur (GE Discovery PET/CT Elite; GE Healthcare, Milwaukee, WI). A 3-dimensional PET model was used, with a matrix of 192 × 192 and an exposure time of 2 min/bed position. Low-dose spiral CT was performed at 120 to 140 kV and 80 ma. After CT attenuation correction, PET images were reconstructed using the algorithm of time-of-flight and point-spread-function, including 2 iterations and 24 subsets.

We used the Advantage Workstation 4.6, equipped with PET Volume Computed Assisted Reading software (PET VCAR; GE Healthcare, Milwaukee, WI) to measure PET metabolic parameters. Two nuclear medicine doctors with more than 15 years of experience independently evaluated and measured the radiologic information using a blinded method. In case of disagreement, another senior doctor was consulted to render a final decision. The software calculated PET parameters using the iterative adaptive algorithm (18), which automatically determines the thresholds for delineating the tumor edge and regions of interest.

Artificial Intelligent Kit software (A.K. 2017; GE Healthcare, Milwaukee, WI) was used for image processing, including preprocessing to homogenize the PET image, and for extracting radiomic features from the PET images according to the artificial sketching area. A total of 254 first-order or higher radiomic features (**Appendix 1**) were extracted for analysis, including gray-level frequency distribution from histogram analysis, the gray-level size zone matrix, the gray-level runlength matrix, and the gray-level co-occurrence matrix (GLCM). Statistical models were applied to these radiomic features to establish a predictive model for the presence of Lynch syndrome. Cohort 1 was randomly divided into a training group (70 patients) and a test group (30 patients) to verify and test the ability of radiomic features to distinguish endometrial cancer related to Lynch syndrome from nonsyndromic endometrial cancer (**Appendix 2**).

### Statistical Analysis

SPSS statistical software (version 21.0; IBM) and MedCalc Statistical Software version 15.2.2 (MedCalc Software bvba, Ostend, Belgium; http://www.medcalc.org; 2015) were used for all analyses. The chi-squared test was used to compare differences in clinical characteristics between patients with the 2 types of endometrial cancer. The Mann-Whitney U test was used to test for non-normally distributed data; normal distribution was tested for using the Kolmogorov-Smirnov test or the Shapiro-Wilk test. Receiver operating characteristic (ROC) analysis was used to test the diagnostic performance of different parameters. We used the Youden index to determine the best cutoff values for radiomic feature. At the same time, calibration curve and decision curve analysis (DCA) were used for evaluating the model. The Spearman correlation coefficient was used to describe the degree of direct correlation of 2 variables.

## Results

In cohort 1, there were 100 eligible patients from January 2008 to October 2019. In cohort 2, there were 23 patients with solid tumors. (**Table 1**)

**Table 1:** Clinical characteristics of patients.

Clinical features	Value
cohort 1	
No. of patients	100
Mean age (95% CI)	56.56(55.06 - 58.06) years
FIGO stage:	
I	68(68%)
II	23 (23%)
III	9 (9%)
Differentiation grade:	
Well differentiated	29(29%)
Moderately differentiated	45(45%)
Poorly differentiated	26(26%)
Histotype:	
Endometrioid	82(82%)
Mixed	6(6%)
Others	12(12%)
Cervical stromal invasion depth:	
< 1/2	61(61%)
≥1/2	39(39%)
cohort 2	
No. of patients	23
Type:	
Squamous cell carcinoma of head and neck	5(22%)
Carcinoma of the lungs (Adenocarcinoma)	4 (17%)
Carcinoma of the lungs (Squamous cell carcinoma)	9 (39%)
Endometrial carcinoma	5(22%)

FIGO, International Federation of Gynecology and Obstetrics.

### PET/CT Parameters and Radiomics in Cohort 1

**Figure 1:**
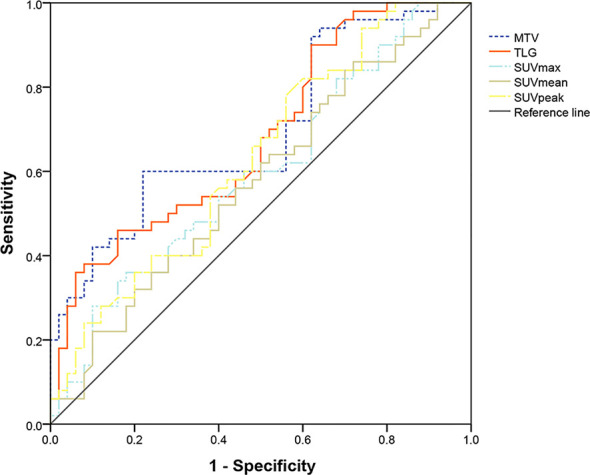
Determination of ROC curve of endometrial cancer with and without Lynch syndrome by PET/CT parameters.

The PET parameters were compared between patients with endometrial carcinoma associated with Lynch syndrome and those with endometrial carcinoma without Lynch syndrome (**Table 2**). The ROC curve for endometrial cancer with or without Lynch syndrome measured by PET/CT parameters shows the predictive value of different PET parameters (**Figure 1**). For metabolic tumor volume (MTV), the best cutoff threshold was 15.82, the area under the curve was 0.695, sensitivity was 60%, and specificity was 78%. For total lesion glycolysis (TLG), the best cutoff threshold was 278.04, the area under the curve was 0.682, sensitivity was 38%, and specificity was 92%.

**Table 2:** The difference of PET/CT parameters and radiomics in cohort 1.

	With Lynch	Without Lynch	P	test
N	50	50		
SUV_max_ (g/cm^3^)	17.92(14.25,21.99)	16.26(13.46,19.88)	0.099	U-test
SUV_mean_ (g/cm^3^)	9.80 (7.78,11.59)	8.99 (5.97,10.95)	0.182	U-test
SUV_peak_(g/cm^3^)	14.42(11.63,18.3)	12.49(9.16,15.88)	0.026	U-test
MTV (cm^3^)	17.88 (7.24,32.77)	11.91 (4.59,15.63)	<0.005	U-test
TLG (g)	143.55(67.48,359.85)	99.96(32.01,163.16)	<0.005	U-test
GLCMEntropy_angle0	10.84(10.09,11.65)	9.29(9.07,9.54)	<0.001	U-test
GLCMEntropy_angle45	10.86(9.97,11.56)	9.13(8.78,9.32)	<0.001	U-test
GLCMEntropy_angle90	10.84(10.09,11.59)	9.24(8.99,9.52)	<0.001	U-test
GLCMEntropy_angle135	10.71(9.98,11.54)	9.2 (8.77,9.46)	<0.001	U-test
GLCMEntropy*	10.74(10.01,11.59)	9.2 (8.91,9.45)	<0.001	U-test

Normal distribution data: means ± standard deviations; t-test.

Non-normal distribution data: medians and interquartile ranges; U-test.

*GLCMEntropy= (GLCMEntropy_angle0 + GLCMEntropy_angle45 + GLCMEntropy_angle90 +GLCMEntropy_angle135)/4.

Of the first- and higher-order radiomic features, only GLCM entropy (GLCMEntropy) from different angles showed a significant difference between the 2 types of endometrial cancer (**Table 2**; p < 0.001). Using the data extracted from the image, we followed the basic idea of the logistic regression to form the ROC curve and the final expression.

(1-1) $$p_{\left(y\right)}=\frac{1}{1+\frac{1}{e^{3.171\;x-30.977}}}$$

We established a probability formula (1-1), using radiomic features, for predicting the presence of Lynch syndrome in patients with endometrial cancer:

Here, P(y) is the probability of Lynch syndrome in patients with endometrial carcinoma, and X is the value of GLCMEntropy in the lesion area on dimensionless PET imaging.

**Figure 2:**
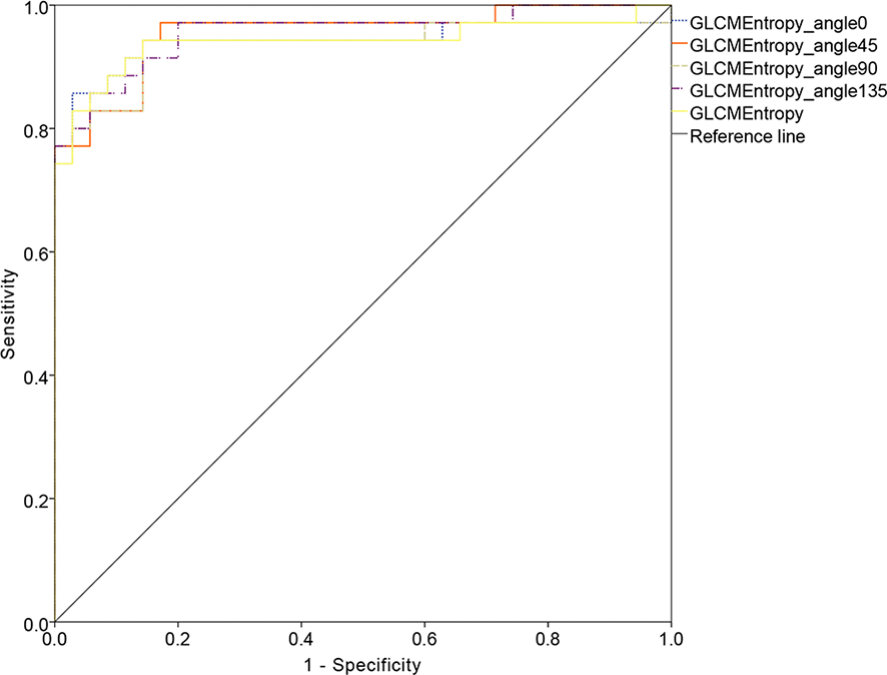
The ROC curve of training group.

**Figure 3:**
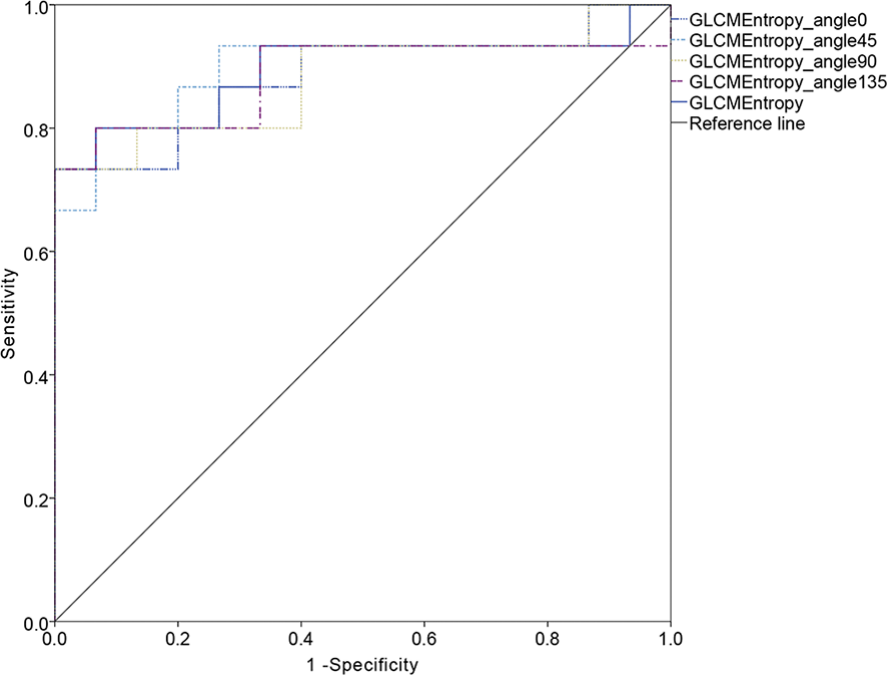
The ROC curve of test group.

**Figure 4:**
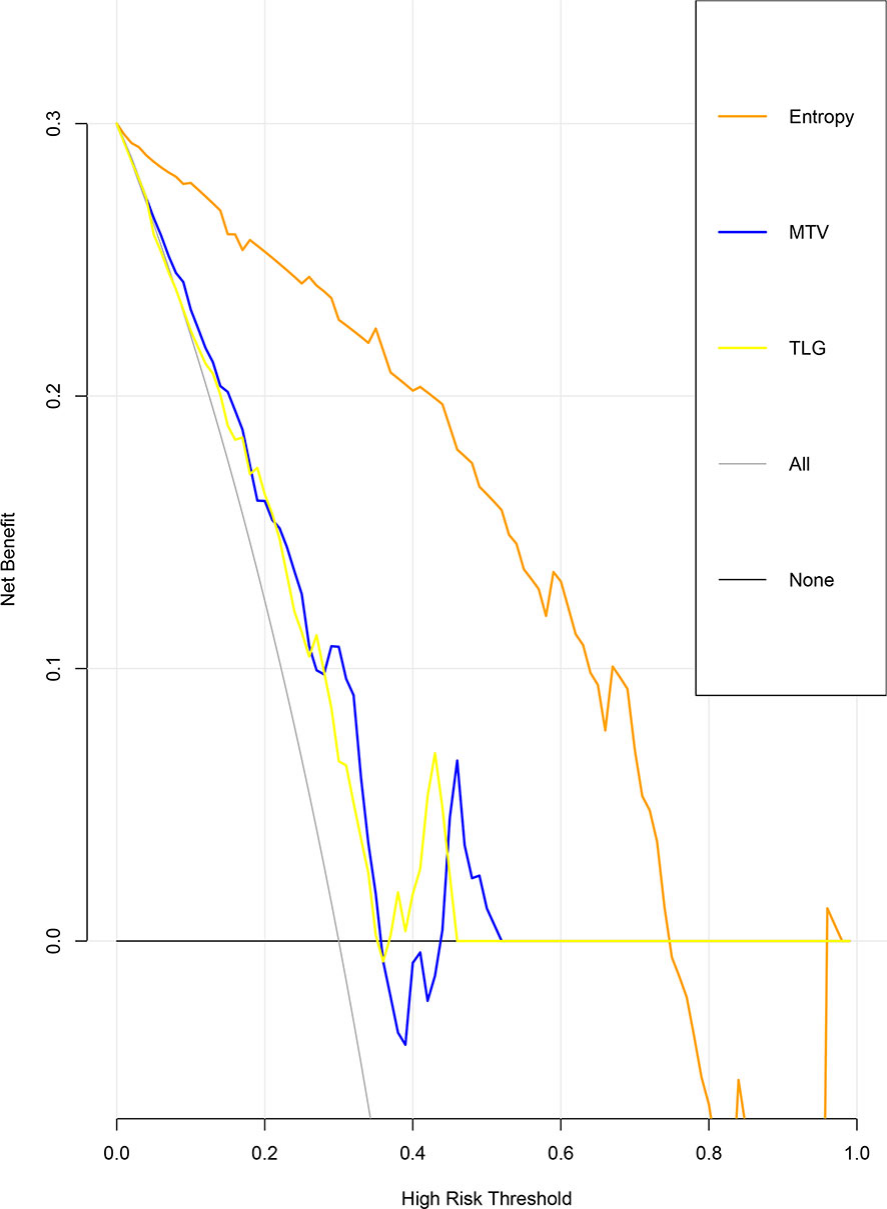
Decision curve analysis between MTV, TLG and GLCMEntropy.

**Figure 5:**
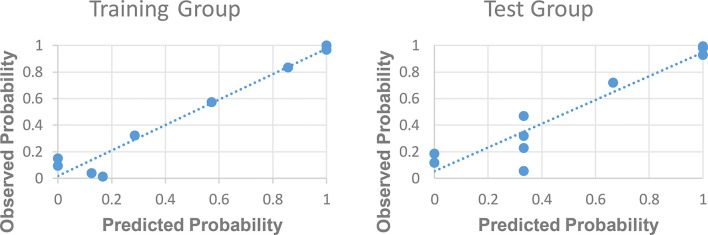
Calibration curve in training group and test group.

The ROC curve for patients with endometrial cancer with or without Lynch syndrome measured by radiomic features demonstrated the resolution of different PET parameters in the training group (**Figure 2**) and the test group (**Figure 3**). The AUC value in the training group was 0.94, and the AUC value in the test group was 0.893. In DCA, we could find GLCMEntropy performs better than MTV and TLG (**Figure 4**). In calibration curve (**Figure 5**), training group and the test group both perform well by Hosmer and Lemeshow test (*p *> 0.05)

### Radiomics (GLCMEntropy) in Cohort 2

**Figure 6:**
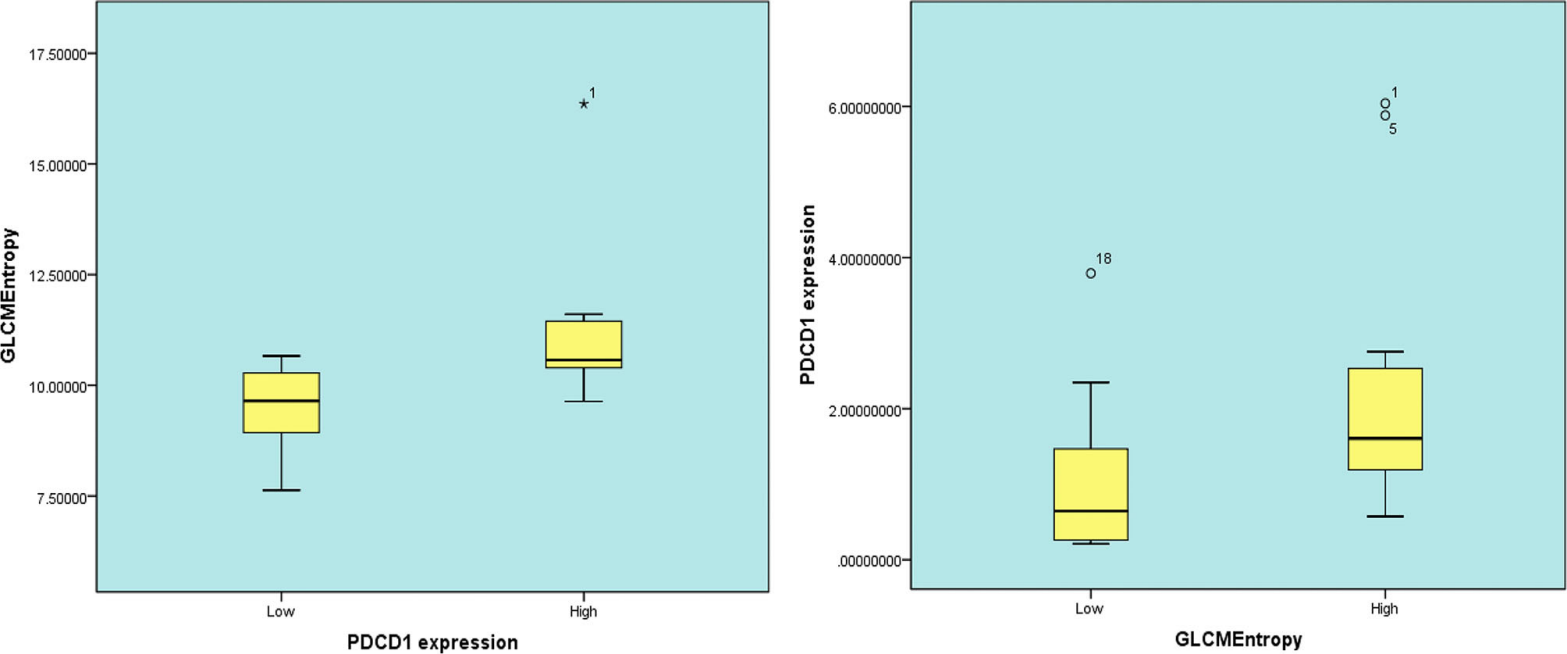
Box diagrams between radiomics and PDCD1 expression. * representing outliers.

**Figure 7:**
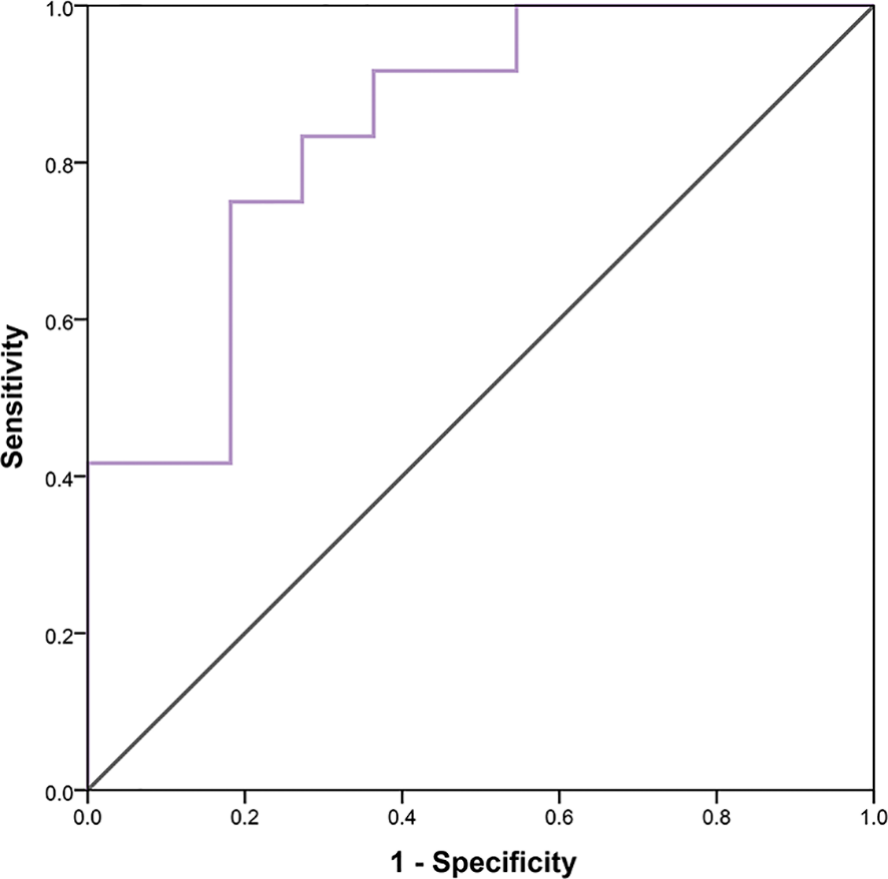
Determination of ROC curve of PDCD1 expression by radiomics.

In 23 patients with solid tumors, the median (SE) value of GLCMEntropy was 10.381 ± 0.340 and of PDCD1 expression was 1.667 ± 0.341. The correlation coefficient between GLCMEntropy and PDCD1 expression was 0.577 (p < 0.001). For the purposes of this presentation, we define herein the low group as those with values less than the median and the high group as those with values equal to or higher than the median (**Figure 6**). We found that PDCD1 expression was 2.346 ± 0.570 in the high GLCMEntropy group, and 1.045 ± 0.320 in the low group (p = 0.06). Conversely, GLCMEntropy was 11.179 ± 0.505 in the high-expression PDCD1 group, and 9.511 ± 0.285 in the low-expression group (p = 0.023). The ROC curve for high and low PDCD1 expression showed potential for predicting PDCD1 expression using the GLCMEntropy value (**Figure 7**). The area under the curve was 0.841, the best cutoff threshold was 9.761, sensitivity was 91.7%, and specificity was 63.6% (*p* = 0.006).

## Discussion

To the best of our knowledge, this work may be the first time to find the significance of PET/CT metabolic parameters and radiomics in the diagnosis of Lynch syndrome in patients with endometrial cancer. Endometrial cancer can be diagnosed using biopsy or postoperative pathology, among other conventional pathologic means, but the diagnosis of Lynch syndrome is not so easy to make. Family history and molecular analysis are the main diagnostic methods used. Compared with CT and magnetic resonance imaging, PET is more sensitive for detecting metastases of endometrial cancer (19) and is often used preoperatively for this purpose. For patients with endometrial cancer, the analysis of PET parameters or radiomic features is focused on detecting lymph node metastasis (20), prognostic value (21) and tissue indentification (18). To our knowledge, this is the first study to evaluate whether FDG PET/CT can be used to identify Lynch syndrome in patients with endometrial cancer.

Radiomic features are able to predict tumor immune infiltration (14, 22) and PD1 expression (23–25), and some studies have shown that they can reflect the efficacy of immunotherapy (26). In this study, we found that endometrial tumors in patients with and without Lynch syndrome have dramatic differences in PD1 expression, and we were able to verify this finding at the gene level. Therefore, we believe that radiomic features have the potential to become a predictor of molecular expression or immunotyping; these conclusions are in line with those of previous studies (22–25).

In Cohort 1, the MTV value was higher in patients with endometrial cancer who had Lynch syndrome. Cosgrove et al (11) found that the tumor volume (pathologic volume) in patients with MMR-deficient endometrial cancer is larger than in those without this deficiency; these results are consistent with ours. It has been reported that the proportion of MMR defects is higher in tumors of larger volume in patients with endometrial cancer (27, 28), a finding that is consistent with the increased MTV we observed in patients with Lynch syndrome. Volumetric parameters, including MTV and TLG, can demonstrate metabolic activity for the total tumor volume. One study (29) found that the absence of MMR is closely related to increased levels of aldolase B protein (one of the catalytic enzymes for glycolysis) and mRNA. Increased values for TLG may be related to the higher malignancy potential of endometrial cancer in patients with Lynch syndrome. Cohort studies (11, 12) have demonstrated that recurrence-free survival in patients with endometrial cancer and Lynch syndrome is significantly reduced compared with those without Lynch syndrome, while other studies found that MTV and TLG can be used as prognostic predictors (21). The levels of MTV and TLG can, to some extent, determine whether patients with endometrial cancer are likely to have Lynch syndrome.

Entropy refers to the regularity of an object: the more ordered, the smaller the entropy. GLCMEntropy represents the degree of heterogeneity, or complexity of texture, in the image in 2 dimensions, which can represent the heterogeneity of the tumor (30). Radiomic study has found that GLCMEntropy is able to describe and evaluate tumor heterogeneity in nasopharyngeal carcinoma (31), breast cancer (32) and esophageal cancer (33). We found that GLCMEntropy is different in patients with and without Lynch syndrome, indicating that the tumor heterogeneity of endometrial cancer in patients with Lynch syndrome is greater than that seen in patients without Lynch syndrome. This may be related to a mutation of MMR genes (e.g., MLH1, MSH2, MSH6, PMS2); the differing mutation rates of these genes result in a variety of variations and molecular phenotypes in various tumor cells (34). We also know that differences in methylation levels of the MLH1 promoter in different parts of the tumor also lead to tumor heterogeneity (35). It has also been shown that immune cell infiltration in the endometrial tumors of patients with Lynch syndrome is higher than that seen in endometrial tumors of patients without Lynch syndrome (8–10); the degree of immune infiltration is closely related to the heterogeneity of the tumor (36).

In Cohort 1 and Cohort 2, we explore the relation between radiomic features and PD1 expression at the protein-expression level and the gene level. The 2 types of endometrial carcinoma included in Cohort 1 could represent PD1-enriched cancer tissue and PD1-deficient cancer tissue (8, 37). The radiomic feature of GLCMEntropy reveals a significant difference in PD1-enriched cancer tissue and PD1-deficient cancer tissue, regardless of the angle used. Using the data from Cohort 2, we found that the radiomic feature of GLCMEntropy and the PDCD1 mRNA levels are strongly correlated. Previous group models have relied on multiparameter and machine-learning methods (38), but a single, robust parameter that is independent of the drawing method has more practical application value: GLCMEntropy is such a parameter (33, 39).

Our study does have some limitations. In Cohort 1, the pathologic sections were not stained to observe immune infiltration. The number of patients analyzed in Cohort 2 is small, which affects the persuasiveness of the results; we expect to obtain a larger sample size in a future prospective study. Finally, radiomics is a new area of study and requires greater standardization and rigorous guidelines as this field of research develops.

## Conclusions

Patients with endometrial cancer who have higher MTV and TLG values are more likely to have Lynch syndrome. A higher value for the radiomic feature of GLCMEntropy, obtained from PET/CT images, indicates that there is a higher risk for Lynch syndrome. The radiomic feature of GLCMEntropy is a potential predictor of PD1 receptor expression, which is valuable for predicting and evaluating the response to immunotherapy.

## Data Availability Statement

In cohort 1, the detailed feature description has been shown in **Appendix 1**, and the specific features can be available through e-mail 995126190@qq.com. In cohort 2, publicly available datasets were analyzed in this study, which can be found at: http://cancerimagingarchive.net, TCIA.

## Ethics Statement

The studies involving human participants were reviewed and approved by Medical ethics committee of Shengjing Hospital of China Medical University. Written informed consent for participation was not required for this study in accordance with the national legislation and the institutional requirements.

## Author Contributions

XW, JJ, and HS: Drafting of the manuscript. KW, YY and XL: Critical revision of the manuscript for important intellectual content. HS: Final approval of manuscript. All authors contributed to the article and approved the submitted version.

## Funding 

This study was funded by LIAONING Science & Technology Project (2017225012), LIAONING Science Natural Science Foundation (2019-MS-373) and 345 Talent Project.

## Conflict of Interest

The authors declare that the research was conducted in the absence of any commercial or financial relationships that could be construed as a potential conflict of interest.

## References

[1] Bray F Ferlay J Soerjomataram I Siegel RL Torre LA Jemal A. Global Cancer Statistics 2018: GLOBOCAN Estimates of Incidence and Mortality Worldwide for 36 Cancers in 185 Countries. CA Cancer J Clin (2018) 68(6):394–424. 10.3322/caac.21492 30207593

[2] Makker V Rasco D Vogelzang NJ Brose MS Cohn AL Mier J. Lenvatinib Plus Pembrolizumab in Patients With Advanced Endometrial Cancer: An Interim Analysis of a Multicentre, Open-Label, Single-Arm, Phase 2 Trial. Lancet Oncol (2019) 20(5):711–8. 10.1016/s1470-2045(19)30020-8 PMC11686814 30922731

[3] Zhang L Kwan SY Wong KK Solaman PT Lu KH Mok SC. Pathogenesis and Clinical Management of Uterine Serous Carcinoma. Cancers (Basel) (2020) 12(3):686. 10.3390/cancers12030686 PMC7140057 32183290

[4] Dillon JL Gonzalez JL DeMars L Bloch KJ Tafe LJ. Universal Screening for Lynch Syndrome in Endometrial Cancers: Frequency of Germline Mutations and Identification of Patients With Lynch-like Syndrome. Hum Pathol (2017) 70:121–8. 10.1016/j.humpath.2017.10.022 29107668

[5] Lancaster JM Powell CB Chen LM Richardson DL. Society of Gynecologic Oncology Statement on Risk Assessment for Inherited Gynecologic Cancer Predispositions. Gynecol Oncol (2015) 136(1):3–7. 10.1016/j.ygyno.2014.09.009 25238946

[6] Lynch HT Snyder CL Shaw TG Heinen CD Hitchins MP. Milestones of Lynch Syndrome: 1895-2015. Nat Rev Cancer (2015) 15(3):181–94. 10.1038/nrc3878 25673086

[7] Hampel H Bennett RL Buchanan A Pearlman R Wiesner GL. A Practice Guideline From the American College of Medical Genetics and Genomics and the National Society of Genetic Counselors: Referral Indications for Cancer Predisposition Assessment. Genet Med (2015) 17(1):70–87. 10.1038/gim.2014.147 25394175

[8] Kim J Kong JK Yang W Cho H Chay DB Lee BH. Dna Mismatch Repair Protein Immunohistochemistry and MLH1 Promotor Methylation Testing for Practical Molecular Classification and the Prediction of Prognosis in Endometrial Cancer. Cancers (Basel) (2018) 10(9):279. 10.3390/cancers10090279 PMC6162750 30134578

[9] Yamashita H Nakayama K Ishikawa M Nakamura K Ishibashi T Sanuki K. Microsatellite Instability is a Biomarker for Immune Checkpoint Inhibitors in Endometrial Cancer. Oncotarget (2018) 9(5):5652–64. 10.18632/oncotarget.23790 PMC5814165 29464025

[10] Pakish JB Zhang Q Chen Z Liang H Chisholm GB Yuan Y. Immune Microenvironment in Microsatellite-Instable Endometrial Cancers: Hereditary or Sporadic Origin Matters. Clin Cancer Res (2017) 23(15):4473–81. 10.1158/1078-0432.Ccr-16-2655 PMC5540763 28264871

[11] Cosgrove CM Cohn DE Hampel H Frankel WL Jones D McElroy JP. Epigenetic Silencing of MLH1 in Endometrial Cancers is Associated With Larger Tumor Volume, Increased Rate of Lymph Node Positivity and Reduced Recurrence-Free Survival. Gynecol Oncol (2017) 146(3):588–95. 10.1016/j.ygyno.2017.07.003 PMC5601318 28709704

[12] Nagle CM O’Mara TA Tan Y Buchanan DD Obermair A Blomfield P. Endometrial Cancer Risk and Survival by Tumor MMR Status. J Gynecol Oncol (2018) 29(3):e39. 10.3802/jgo.2018.29.e39 29533022 PMC5920223

[13] Lin DI Hecht JL. Targeted Screening With Combined Age- and Morphology-Based Criteria Enriches Detection of Lynch Syndrome in Endometrial Cancer. Int J Surg Pathol (2016) 24(4):297–305. 10.1177/1066896916629782 26842347

[14] Sun R Limkin EJ Vakalopoulou M Dercle L Champiat S Han SR. A Radiomics Approach to Assess Tumour-Infiltrating CD8 Cells and Response to anti-PD-1 or anti-PD-L1 Immunotherapy: An Imaging Biomarker, Retrospective Multicohort Study. Lancet Oncol (2018) 19(9):1180–91. 10.1016/s1470-2045(18)30413-3 30120041

[15] Weinstein JN Collisson EA Mills GB Shaw KR Ozenberger BA Ellrott K. The Cancer Genome Atlas Pan-Cancer Analysis Project. Nat Genet (2013) 45(10):1113–20. 10.1038/ng.2764 PMC3919969 24071849

[16] Clark K Vendt B Smith K Freymann J Kirby J Koppel P. The Cancer Imaging Archive (TCIA): Maintaining and Operating a Public Information Repository. J Digit Imaging (2013) 26(6):1045–57. 10.1007/s10278-013-9622-7 PMC3824915 23884657

[17] Lyu X Zhang M Li G Jiang Y Qiao Q. PD-1 and PD-L1 Expression Predicts Radiosensitivity and Clinical Outcomes in Head and Neck Cancer and is Associated With HPV Infection. J Cancer (2019) 10(4):937–48. 10.7150/jca.27199 PMC6400795 30854100

[18] Wang T Sun H Guo Y Zou L. (18)F-Fdg PET/CT Quantitative Parameters and Texture Analysis Effectively Differentiate Endometrial Precancerous Lesion and Early-Stage Carcinoma. Mol Imaging (2019) 18:1536012119856965. 10.1177/1536012119856965 31198089 PMC6572902

[19] Atri M Zhang Z Dehdashti F Lee SI Marques H Ali S. Utility of PET/CT to Evaluate Retroperitoneal Lymph Node Metastasis in High-Risk Endometrial Cancer: Results of ACRIN 6671/Gog 0233 Trial. Radiology (2017) 283(2):450–9. 10.1148/radiol.2016160200 PMC5410939 28051912

[20] Crivellaro C Landoni C Elisei F Buda A Bonacina M Grassi T. Combining Positron Emission Tomography/Computed Tomography, Radiomics, and Sentinel Lymph Node Mapping for Nodal Staging of Endometrial Cancer Patients. Int J Gynecol Cancer (2020) 30(3):378–82. 10.1136/ijgc-2019-000945 32079712

[21] Erdogan M Erdemoglu E Evrimler Ş Hanedan C Şengül SS. Prognostic Value of Metabolic Tumor Volume and Total Lesion Glycolysis Assessed by 18F-FDG PET/CT in Endometrial Cancer. Nucl Med Commun (2019) 40(11):1099–104. 10.1097/mnm.0000000000001091 31568194

[22] Mazzaschi G Milanese G Pagano P Madeddu D Gnetti L Trentini F. Integrated CT Imaging and Tissue Immune Features Disclose a Radio-Immune Signature With High Prognostic Impact on Surgically Resected NSCLC. Lung Cancer (2020) 144:30–9. 10.1016/j.lungcan.2020.04.006 32361033

[23] Sun Z Hu S Ge Y Wang J Duan S Hu C. Radiomics Study for Predicting the Expression of PD-L1 in non-Small Cell Lung Cancer Based on CT Images and Clinicopathologic Features. J Xray Sci Technol (2020) 28(3):449–59. 10.3233/xst-200642 32176676

[24] Aslan K Turco V Blobner J Sonner JK Liuzzi AR Núñez NG. Heterogeneity of Response to Immune Checkpoint Blockade in Hypermutated Experimental Gliomas. Nat Commun (2020) 11(1):931. 10.1038/s41467-020-14642-0 32071302 PMC7028933

[25] Yoon J Suh YJ Han K Cho H Lee HJ Hur J. Utility of CT Radiomics for Prediction of PD-L1 Expression in Advanced Lung Adenocarcinomas. Thorac Cancer (2020) 11(4):993–1004. 10.1111/1759-7714.13352 32043309 PMC7113038

[26] Polverari G Ceci F Bertaglia V Reale ML Rampado O Gallio E. (18)F-Fdg Pet Parameters and Radiomics Features Analysis in Advanced Nsclc Treated With Immunotherapy as Predictors of Therapy Response and Survival. Cancers (Basel) (2020) 12(5):1163. 10.3390/cancers12051163 PMC7281558 32380754

[27] Kandoth C Schultz N Cherniack AD Akbani R Liu Y Shen H. Integrated Genomic Characterization of Endometrial Carcinoma. Nature (2013) 497(7447):67–73. 10.1038/nature12113 23636398 PMC3704730

[28] McConechy MK Talhouk A Li-Chang HH Leung S Huntsman DG Gilks CB. Detection of DNA Mismatch Repair (MMR) Deficiencies by Immunohistochemistry can Effectively Diagnose the Microsatellite Instability (MSI) Phenotype in Endometrial Carcinomas. Gynecol Oncol (2015) 137(2):306–10. 10.1016/j.ygyno.2015.01.541 25636458

[29] Lian J Xia L Chen Y Zheng J Ma K Luo L. Aldolase B Impairs DNA Mismatch Repair and Induces Apoptosis in Colon Adenocarcinoma. Pathol Res Pract (2019) 215(11):152597. 10.1016/j.prp.2019.152597 31564566

[30] Forghani R Savadjiev P Chatterjee A Muthukrishnan N Reinhold C Forghani B. Radiomics and Artificial Intelligence for Biomarker and Prediction Model Development in Oncology. Comput Struct Biotechnol J (2019) 17:995–1008. 10.1016/j.csbj.2019.07.001 31388413 PMC6667772

[31] Mao J Fang J Duan X Yang Z Cao M Zhang F. Predictive Value of Pretreatment MRI Texture Analysis in Patients With Primary Nasopharyngeal Carcinoma. Eur Radiol (2019) 29(8):4105–13. 10.1007/s00330-018-5961-6 PMC6610272 30617473

[32] Lemarignier C Martineau A Teixeira L Vercellino L Espié M Merlet P. Correlation Between Tumour Characteristics, SUV Measurements, Metabolic Tumour Volume, TLG and Textural Features Assessed With (18)F-FDG PET in a Large Cohort of Oestrogen Receptor-Positive Breast Cancer Patients. Eur J Nucl Med Mol Imaging (2017) 44(7):1145–54. 10.1007/s00259-017-3641-4 28188325

[33] Hatt M Tixier F Cheze Le Rest C Pradier O Visvikis D. Robustness of Intratumour ¹⁸F-FDG PET Uptake Heterogeneity Quantification for Therapy Response Prediction in Oesophageal Carcinoma. Eur J Nucl Med Mol Imaging (2013) 40(11):1662–71. 10.1007/s00259-013-2486-8 23857457

[34] Haraldsdottir S Rafnar T Frankel WL Einarsdottir S Sigurdsson A Hampel H. Comprehensive Population-Wide Analysis of Lynch Syndrome in Iceland Reveals Founder Mutations in MSH6 and PMS2. Nat Commun (2017) 8:14755. 10.1038/ncomms14755 28466842 PMC5418568

[35] Pai RK Plesec TP Abdul-Karim FW Yang B Marquard J Shadrach B. Abrupt Loss of MLH1 and PMS2 Expression in Endometrial Carcinoma: Molecular and Morphologic Analysis of 6 Cases. Am J Surg Pathol (2015) 39(7):993–9. 10.1097/pas.0000000000000415 25786082

[36] Losic B Craig AJ Villacorta-Martin C Martins-Filho SN Akers N Chen X. Intratumoral Heterogeneity and Clonal Evolution in Liver Cancer. Nat Commun (2020) 11(1):291. 10.1038/s41467-019-14050-z 31941899 PMC6962317

[37] Ono R Nakayama K Nakamura K Yamashita H Ishibashi T Ishikawa M. Dedifferentiated Endometrial Carcinoma Could be A Target for Immune Checkpoint Inhibitors (Anti PD-1/PD-L1 Antibodies). Int J Mol Sci (2019) 20(15):3744. 10.3390/ijms20153744 PMC6696376 31370215

[38] Limkin EJ Sun R Dercle L Zacharaki EI Robert C Reuzé S. Promises and Challenges for the Implementation of Computational Medical Imaging (Radiomics) in Oncology. Ann Oncol (2017) 28(6):1191–206. 10.1093/annonc/mdx034 28168275

[39] Orlhac F Soussan M Maisonobe JA Garcia CA Vanderlinden B Buvat I. Tumor Texture Analysis in 18F-FDG PET: Relationships Between Texture Parameters, Histogram Indices, Standardized Uptake Values, Metabolic Volumes, and Total Lesion Glycolysis. J Nucl Med (2014) 55(3):414–22. 10.2967/jnumed.113.129858 24549286

