# Supplementary material for: Additional Value of PET/CT-Based Radiomics to Metabolic Parameters in Diagnosing Lynch Syndrome and Predicting PD1 Expression in Endometrial Carcinoma
Source: Front Oncol. 2021 May 12;11:595430. doi: 10.3389/fonc.2021.595430 (PMC8152935; doi:10.3389/fonc.2021.595430)
Supplement: Supplementary file 1 [file DataSheet_1.docx]

**Appendix 2**

Cohort 1 was randomly divided into a training group (70 patients) and a test group (30 patients) to verify and test the ability of radiomic features for identifying Lynch syndrome in patients with endometrial cancer. Our method was random sampling, which ensured the randomness and reliability of grouping results. Through statistical analysis, there was no statistical difference in clinical characteristics between the two groups (*p*﹤0.05).

**Appendix Table 1: Clinical characteristics of training group and test group**

| Clinical features | Value | |
| --- | --- | --- |
|  | training group | test group |
| No. of patients | 70 | 30 |
| Mean age (95% CI) | 55.97(54.57 - 57.37) years | 56.84(55.24 - 58.44) years |
| FIGO stage: |  |  |
| I | 47(69%) | 21(31%) |
| II | 15(65%) | 8(35%) |
| III | 6(67%) | 3(33%) |
| Differentiation grade: |  |  |
| Well differentiated | 22(76%) | 7(24%) |
| Moderately differentiated | 30(67%) | 15(33%) |
| Poorly differentiated | 17(65%) | 9(35%) |
| Histotype: |  |  |
| Endometrioid | 52(63%) | 30(37%) |
| Mixed | 3(50%) | 3(50%) |
| Others | 7(58%) | 5(42%) |
| Cervical stromal invasion depth: |  |  |
| < 1/2 | 39(64%) | 22(36%) |
| ≥1/2 | 26(67%) | 13(33%) |

FIGO, International Federation of Gynecology and Obstetrics.
