# Supplementary material for: Additional Value of PET/CT-Based Radiomics to Metabolic Parameters in Diagnosing Lynch Syndrome and Predicting PD1 Expression in Endometrial Carcinoma
Source: Front Oncol. 2021 May 12;11:595430. doi: 10.3389/fonc.2021.595430 (PMC8152935; doi:10.3389/fonc.2021.595430)
Supplement: Supplementary file 2 [file Presentation_1.pptx]

## Slide 1
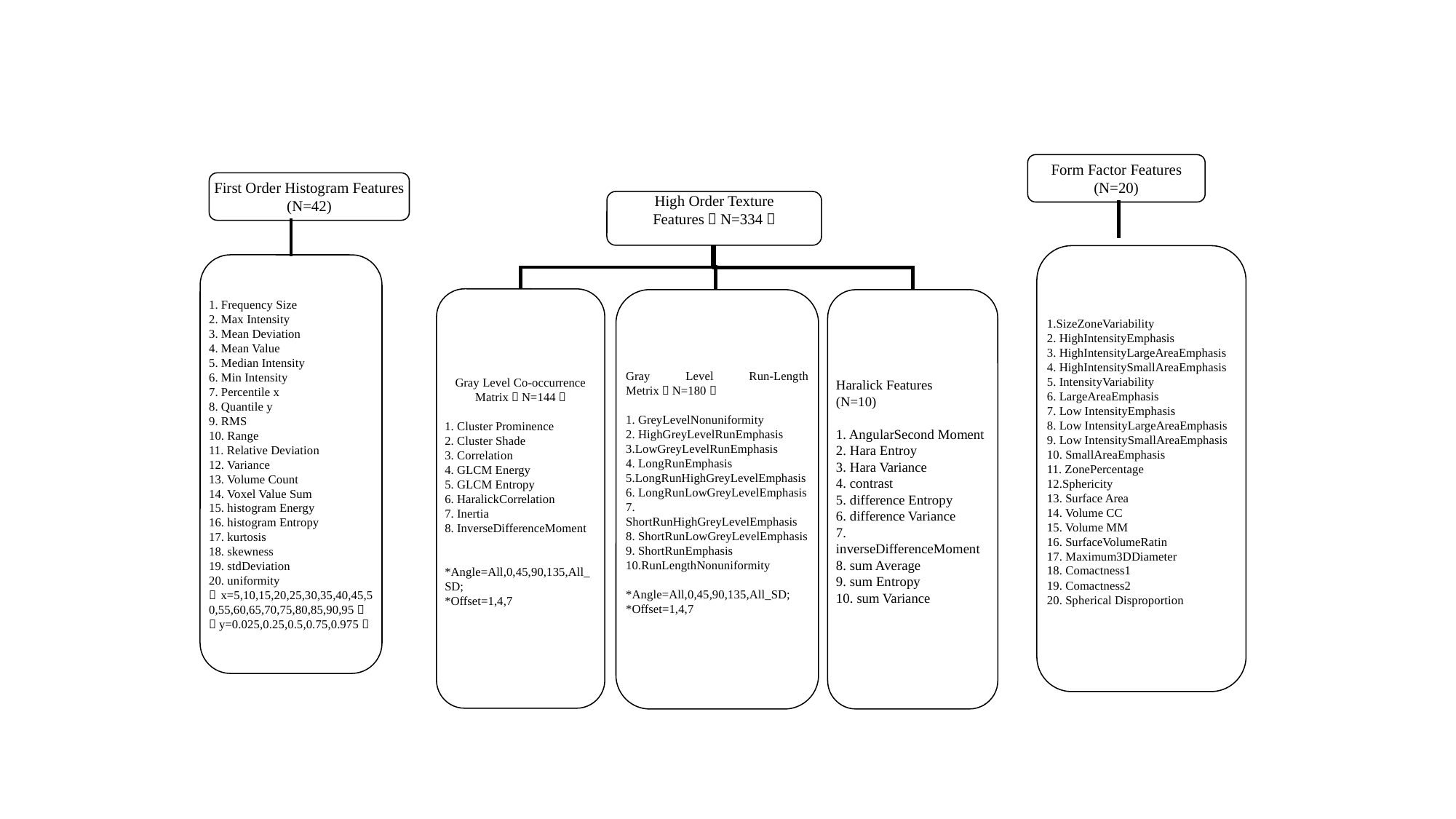

High Order Texture Features（N=334）
Gray Level Co-occurrence Matrix（N=144）
1. Cluster Prominence
2. Cluster Shade
3. Correlation
4. GLCM Energy
5. GLCM Entropy
6. HaralickCorrelation
7. Inertia
8. InverseDifferenceMoment
*Angle=All,0,45,90,135,All_SD;
*Offset=1,4,7
Gray Level Run-Length Metrix（N=180）
1. GreyLevelNonuniformity
2. HighGreyLevelRunEmphasis
3.LowGreyLevelRunEmphasis
4. LongRunEmphasis
5.LongRunHighGreyLevelEmphasis
6. LongRunLowGreyLevelEmphasis
7. ShortRunHighGreyLevelEmphasis
8. ShortRunLowGreyLevelEmphasis
9. ShortRunEmphasis
10.RunLengthNonuniformity
*Angle=All,0,45,90,135,All_SD;
*Offset=1,4,7
Haralick Features
(N=10)
1. AngularSecond Moment
2. Hara Entroy
3. Hara Variance
4. contrast
5. difference Entropy
6. difference Variance
7. inverseDifferenceMoment
8. sum Average
9. sum Entropy
10. sum Variance
Form Factor Features
(N=20)
First Order Histogram Features
(N=42)
1.SizeZoneVariability
2. HighIntensityEmphasis
3. HighIntensityLargeAreaEmphasis
4. HighIntensitySmallAreaEmphasis
5. IntensityVariability
6. LargeAreaEmphasis
7. Low IntensityEmphasis
8. Low IntensityLargeAreaEmphasis
9. Low IntensitySmallAreaEmphasis
10. SmallAreaEmphasis
11. ZonePercentage
12.Sphericity
13. Surface Area
14. Volume CC
15. Volume MM
16. SurfaceVolumeRatin
17. Maximum3DDiameter
18. Comactness1
19. Comactness2
20. Spherical Disproportion
1. Frequency Size
2. Max Intensity
3. Mean Deviation
4. Mean Value
5. Median Intensity
6. Min Intensity
7. Percentile x
8. Quantile y
9. RMS
10. Range
11. Relative Deviation
12. Variance
13. Volume Count
14. Voxel Value Sum
15. histogram Energy
16. histogram Entropy
17. kurtosis
18. skewness
19. stdDeviation
20. uniformity
（x=5,10,15,20,25,30,35,40,45,50,55,60,65,70,75,80,85,90,95）
（y=0.025,0.25,0.5,0.75,0.975）
